# Expression Authenticity: The Role of Genuine and Deliberate Displays in Emotion Perception

**DOI:** 10.3389/fpsyg.2020.611248

**Published:** 2021-01-14

**Authors:** Mircea Zloteanu, Eva G. Krumhuber

**Affiliations:** ^1^Department of Criminology and Sociology, Kingston University London, Kingston, United Kingdom; ^2^Department of Psychology, Kingston University London, Kingston, United Kingdom; ^3^Department of Experimental Psychology, University College London, London, United Kingdom

**Keywords:** emotion, facial expressions, genuine, posed and spontaneous, authenticity discrimination

## Abstract

People dedicate significant attention to others’ facial expressions and to deciphering their meaning. Hence, knowing whether such expressions are genuine or deliberate is important. Early research proposed that authenticity could be discerned based on reliable facial muscle activations unique to genuine emotional experiences that are impossible to produce voluntarily. With an increasing body of research, such claims may no longer hold up to empirical scrutiny. In this article, expression authenticity is considered within the context of senders’ ability to produce convincing facial displays that resemble genuine affect and human decoders’ judgments of expression authenticity. This includes a discussion of spontaneous vs. posed expressions, as well as appearance- vs. elicitation-based approaches for defining emotion recognition accuracy. We further expand on the functional role of facial displays as neurophysiological states and communicative signals, thereby drawing upon the *encoding-decoding* and *affect-induction* perspectives of emotion expressions. Theoretical and methodological issues are addressed with the aim to instigate greater conceptual and operational clarity in future investigations of expression authenticity.

## Introduction

The accurate recognition of emotions plays a crucial role in communication and social interaction. Knowing what another person is feeling is relevant for predicting their psychological state, likely future behavior, and interaction outcome (Hall et al., 2009). However, the advantage of such knowledge hinges on the emotional displays matching the person’s true underlying affect.

Humans have great control over their facial behavior (Zuckerman et al., 1986; Smith, 2004), with the ability to produce complex expressions. This implies that not all displays genuinely reflect a person’s underlying emotional state (Barrett, 2006). Deliberate expressions reflect the strategic intent of the individual in the absence of felt emotions (Ekman and Rosenberg, 2005). During social interaction, individuals may consciously regulate and suppress their emotions and portray expressions of unfelt emotions. This raises the issue of *expression authenticity*.

While people seem capable of recognizing emotions from specific facial configurations with high accuracy (Calvo and Nummenmaa, 2015),^1^ the ability to distinguish the authenticity of such expressions is much poorer (Ekman and O’Sullivan, 1991; Hess and Kleck, 1994; McLellan et al., 2010). Interestingly, the reason(s) for this has not been fully determined yet, with difficulties in explanations partly stemming from disagreements about the nature of emotions and the function of facial expressions. Recent propositions have attempted to elucidate some of the inconsistencies of past research, considering facial expressions as both *innate cues* and *communicative signals* (Crivelli and Fridlund, 2018; Barrett et al., 2019). Here, we build on this work, thereby focusing on human expression authenticity judgments: assessing whether the emotional expression displayed by another person is a genuine reflection of their underlying affect. This operational definition is representative of the task participants typically perform and the instructions they receive; however, as will be discussed, how one conceptualizes emotions and operationalizes facial expressions will ultimately determine what an authenticity judgment indicates and the inferences that can be drawn from it.^2^

## The Function of Facial Expressions: Encoding-Decoding vs. Affect-Induction

Conceptually, there are two main perspectives regarding the function of facial expressions. These view facial displays either as (a) innate cues reflecting genuine affect or (b) communicative signals of affect and intent.

According to the *encoding-decoding* perspective (Ekman, 2003), observers (called *decoders*) “decode” the meaning behind emotional displays of others (called *senders*). Facial expressions are considered to be innate, neurologically activated, fixed facial muscle patterns that occur in response to emotion-eliciting stimuli (Tomkins, 1962). Their appearance is an evolved response to specific events that are difficult (if not impossible) to suppress (Hurley and Frank, 2011), resulting in facial *leakage* (i.e., involuntary displays of felt emotions; Ekman, 1997). As such, they are functionally not a source of emotional information but became so as an exaptation (Darwin, 1872). Under this account, voluntary modulations of expressions come in the form of *display rules*, which are socio-cultural norms regulating the expression of displays (Ekman and Friesen, 1971). For expression authenticity, this perspective places emphasis on presumed *reliable muscles*, which are facial markers said to activate only during felt affect and being impossible to voluntarily control (Ekman, 2003). Under this view, differences between genuine and deliberate displays exist, and expression authenticity is a function of the decoder’s perceptual ability and knowledge for making accurate inferences. While being popular, this view has been criticized due to its vague conceptualization and lack of empirical support (Barrett et al., 2019).

According to the *affect-induction* perspective (Crivelli and Fridlund, 2018, 2019),^3^ facial displays function as a signaling mechanism to communicate one’s emotional states, motivating corresponding states in the observer (called *receivers*). Evolutionary there is no reason why facial expressions and emotion perception could not have co-evolved as part of a social signaling system (Izard, 1994; Dezecache et al., 2013). Indeed, a growing body of evidence suggests that humans are adept at producing facial expressions for communicative reasons (Smith, 2004). Under this view, the function of emotional displays is to signal emotional information and intent (i.e., they are not cryptic “cues” needed to be decoded; Crivelli and Fridlund, 2019). This perspective is not without its limitations. For instance, there are clear examples of behavior, such as blushing (Crozier, 2010), which can be used to infer the emotional states the sender may wish to suppress but is unable to do so. Also, the perspective does not adequately account for emotional leakage or solitary reactions (e.g., smiling when alone; but see Crivelli and Fridlund, 2018).

When synthesized both perspectives are useful for understanding human expression authenticity judgments. For instance, the encoding-decoding perspective provides the foundation for considering genuine (i.e., innate, involuntary responses) and deliberate (i.e., voluntary, communicative signals) expressions. It is important to note, though, that the argument for clear differences in expression authenticity (Ekman et al., 1988) is neither consistent with empirical investigations (Barrett et al., 2019) nor reflected in human judgments of facial expressions (Zloteanu et al., 2020, 2018). First, senders seem to possess the ability to produce genuine-looking displays of emotion (Surakka and Hietanen, 1998; Gosselin et al., 2010; Gunnery et al., 2013). Second, when considering facial expressions as social signals, as done in the affect-induction perspective, it is possible to understand why expression authenticity judgments are relatively poor. In deceptive scenarios, deliberate emotional cues serve an obvious communicative purpose: they convey an affective state to an observer which strategically benefits the sender (maliciously or otherwise).

## Spontaneous vs. Posed: A Sufficiently Nuanced Dichotomy?

Irrespective of the perspective adopted, researchers typically employ an experimental design that separates facial expressions into *spontaneous* and *posed*. These are generated in various ways, ranging from emotion-induced exemplars to directed facial muscle activations (Coan and Allen, 2007; Quigley et al., 2013; Siedlecka and Denson, 2019). The conceptualization, however, has been criticized for not reflecting the nuances in expression elicitation (Shackman and Wager, 2019).

Proponents of the encoding-decoding perspective treat spontaneous and posed displays as categorical, with “spontaneous” reflecting felt emotional displays and “posed” reflecting unfelt deliberate displays. The origin of this dichotomy stems from neuroanatomical research alleging separate neural pathways for the production of involuntary and voluntary facial expressions (Rinn, 1984). The two systems are argued to produce visible differences in facial muscle activation, intensity, facial symmetry, and dynamics (Ekman, 2003). Yet, these have been challenged in recent work. For example, research finds no strong support for reliable muscles in either laboratory (Krumhuber and Manstead, 2009) or naturalistic studies (Fernández-Dols and Crivelli, 2013). Also, intensity relates more to the production method than to veracity (Zloteanu et al., 2020, 2018; Miller et al., 2020), and differences in the dynamic components are found to be subtle and varied between emotions (Cohn and Schmidt, 2004; Namba et al., 2016).

Proponents of the affect-induction perspective treat spontaneous and posed as one dimension of emotional displays. The use of actors, for instance, has been proposed to be a valid approach for studying expression authenticity (Gur et al., 2002). Proponents of actor portrayals argue that unmodulated and authentic expressions absent of socio-cultural influence are rare and difficult (if not impossible) to elicit under laboratory conditions (Scherer and Bänziger, 2010). The use of actors permits the creation of well-controlled, reliable, and recognizable displays to investigate the “shared code of emotional signalling” (Scherer and Bänziger, 2010, p. 166); although, the specific acting technique may play a similarly important role (Orlowska et al., 2018; Krumhuber et al., 2020). Nonetheless, such research has often been criticized due to the intentional communicative nature of portrayals, arguing that the reliance on actors for both spontaneous and posed displays invalidates the concept of authenticity (Cowie et al., 2005; Sauter and Fischer, 2018). We conjecture that the use of actors raises an interesting theoretical point. If actors can reproduce elements of genuine, spontaneous, felt displays (e.g., Carroll and Russell, 1997), it calls into question whether authenticity discrimination as a perceptual ability is possible *per se*.

Ultimately, terms such as “genuine” and “spontaneous” should be treated with caution as—theoretically and methodologically—they are debatable concepts. While some researchers treat them as synonyms, others consider them as different dimensions (i.e., genuine-deceptive and spontaneous-posed). For encoding-decoding scholars, genuine and spontaneous reflect similar concepts, namely, the absence of modulation and intentionality in the emotional display (Ekman, 2003). Yet, for affect-induction scholars the genuine-deceptive dimension reflects the intent of the sender (Crivelli and Fridlund, 2018), while spontaneous/posed are labels given to displays with specific facial characteristics. It is important to note that emotional congruence and sender control are complex issues. For instance, an expression may match the person’s emotional state but be deliberately produced, such as exaggerated displays (e.g., laughing more strongly in the presence of others; Fridlund, 1991). Based on emotional congruence this would be considered as genuine (and potentially even spontaneous); yet, based on control it can be labeled as deceptive (and posed). Careful considerations should also be given to the type of expression as there are many ways of eliciting either spontaneous or posed displays (Zloteanu et al., 2018, 2020; Krumhuber et al., 2019).

## How Do We Measure “Accuracy”: Appearance-Based or Elicitation-Based?

Emotional experiences are often difficult to measure, with some scholars even arguing that they are empirically unverifiable (i.e., we can never truly know what someone is experiencing). Most investigations rely on proxies such as self-report or bodily measures (for recent commentaries see Barrett et al., 2019; Crivelli and Fridlund, 2019). This begs the question: what do we mean by “accurate emotion recognition?”

A review of the literature reveals multiple processes with similar yet not equivalent terms and definitions, such as emotion *identification*, *categorization*, *discrimination*, *inference*, and *recognition*. These are used interchangeably or separately, and sometimes the same term has different definitions (see Gonçalves et al., 2018), making it difficult to know if two scholars pertain to the same phenomenon. For instance, in our research (Zloteanu et al., 2020, 2018) we define *emotion classification accuracy* as the ability to infer specific emotions from facial displays, and *emotion authenticity discrimination* as the ability to differentiate between spontaneous (genuine) and posed (deliberate) displays. By contrast, Buck et al. (2017) use the exact opposite definitions which they label *emotion categorization ability* and *emotion communication accuracy*. Such interchangeable use in terminology may lead to confusion or misleading conclusions and interfere with attempts to synthesize research (see Fiske, 2020). This is a symptom of a larger issue within psychology relating to the use of operational definitions to explain phenomena (see Lilienfeld et al., 2015).

Much of the expression authenticity research has employed an *appearance-based* approach, thereby focusing on stimulus features, such as the Duchenne marker for the distinction between genuine and deliberate smiles. Appearance-based approaches make strong assumptions for the presence/absence of specific facial markers and dynamic features (Ekman, 2003) and impose constraints as to which exemplars are representative of authenticity (thereby excluding facial responses if they fail to meet relevant criteria). Under this approach, judges engage in a categorization task that prompts them to classify facial exemplars based on pre-selected criteria (e.g., Ekman et al., 1983). While such procedure allows for clear and reliable assessments, it may not be sufficient for measuring expression authenticity, as investigations can be conducted with stimuli produced to “look” authentic even though the sender did not experience genuine affect (as often the case with actor portrayals; Scherer and Bänziger, 2010). As such, it only assesses the perceptual or categorization ability of the observer.

The *elicitation-based pathway* is an alternative approach that places the focus on the methods used to produce facial expressions. Here, expression authenticity is operationalized on the basis of sender veracity (or intent), where genuine expressions reflect responses to an emotion-evoking event and non-genuine expressions are voluntarily produced displays in the absence of such an event. It makes no assumptions as to what constitutes a veridical emotional display and merely refers to the congruence between the eliciting event and the external behavior. Natural variations in displays between senders are considered relevant for the judgment process by decoders. Exemplars are selected based on the elicitation technique, allowing researchers to explore how differently produced displays affect people’s judgments. Under this approach, labels such as “genuine” and “deliberate” apply only to the inferences made by judges. While elicitation-based approaches introduce more variability in judgments, they capture the diversity of facial displays and mirror the emotional inferences made in real life. This is in stark contrast to the appearance-based approach in which facial exemplars must adhere to a strict morphological or dynamic criterion regardless of the production method being used.

Both approaches have merits yet answer different questions. The appearance-based approach permits investigations of universal representations of expressions (i.e., prototypical displays), in decoders’ ability to detect specific facial configurations, and how alterations of such patterns impact perception and judgment. The elicitation-based approach permits investigations of the variability in human responses to emotional events, how such behaviors are affected by context or experimental manipulation, and how people infer meaning from such displays. Noteworthy, measures of accuracy have a different meaning under the two approaches. According to the latter, judgment accuracy is more akin to *congruency* (as in Dawel et al., 2017), where a judgment is correct if the expression is judged as “genuine” and the sender was experiencing an emotion. By contrast, appearance-based accuracy reflects the correct identification and grouping of expressions with similar facial patterns irrespective of the sender’s affective experience.

## Recommendations for Future Research

For future investigations of expression authenticity, we recommend the use of advanced statistical analyses, such as Bayesian mixed-effects models, to account for individual differences in senders and judges as sources for variability (see Sorensen et al., 2016). For example, a study comparing genuine and deliberate expressions may find no overall difference in genuineness ratings, yet inspection of the stimuli reveals that some expressions in the deliberate condition were rated overly genuine, thereby influencing the aggregate score. Omitting those expressions as “bad” exemplars may be unjustifiable as one would need to assume the existence of “good” exemplars; instead, the respective senders may have just been excellent actors who produced convincing portrayals. In a similar vein, some observers may systematically underrate genuine expressions, minimizing potential differences between conditions. Using mixed-effects models such variability can easily be accounted for without the need to remove data or make assumptions regarding its validity, thereby allowing for more robust analyses (Brysbaert and Stevens, 2018).

Separate from the sender-judge variability within and between studies, considerations should be given to biases in authenticity judgments. For sender-specific biases, the *demeanor bias* – the finding that some senders produce general impressions of (dis)honesty irrespective of their veracity (Zuckerman et al., 1981; Levine et al., 2011) – plays an important role. A person’s demeanor may result in their display being judged as non-genuine, irrespective of appearance, or intent. Merely examining facial features (i.e., Duchenne marker) will not reveal such perceptual biases toward particular senders. For judge-specific biases, response tendencies such as the *truth-bias* may impact expression judgments. Overestimating others’ truthfulness results in inflated accuracy scores which do not reflect true detection ability but a response preference (see Zuckerman et al., 1981). People may be biased toward disproportionally assuming that facial expressions are genuine (i.e., *authenticity bias*; Gosselin et al., 1995; Zloteanu, 2020). Hence, it is crucial to separate response biases from signal detection when measuring accuracy (Stanislaw and Todorov, 1999).

Future research should also embrace the wide range in which facial expressions occur. Studies concerned with authenticity typically employ one set of spontaneous and posed stimuli, pre-selected from many exemplars and based on specific criteria (see Krumhuber et al., 2017). Rarely do investigations target multiple types of displays (e.g., Soppe, 1988). Given that “spontaneous” and “posed” serve as umbrella terms (see Sauter and Fischer, 2018; Siedlecka and Denson, 2019), judgments under one operationalization may not generalize to another, and aggregating findings will result in incorrect and misleading inferences. In Zloteanu et al. (2018, 2020), we illustrated how producing deliberate expressions using different methods results in judgment differences for each expression type. Under a classical one genuine vs. one deliberate design, these results would not be easily interpretable as each comparison produces different insights into expression authenticity judgments.

Finally, it would be desirable to aim for greater transparency and consistency in the use of operational definitions, urging researchers to be explicit, comprehensive, and transparent in their methodology. While some scholars may be aware of the nuances and shortcomings of specific terminology (Barrett et al., 2019), over-labeling measures and phenomena increase the risk of confusion within and across a domain (Lilienfeld et al., 2015). Labels should serve as mere conveniences for scientific communication, but do not represent unchallengeable and unfalsifiable constructs. Given that emotion scholars still debate the exact definition of emotions (Ortony and Turner, 1990; Izard, 2007; Kagan, 2007), their taxonomy (Fiske, 2020), and whether they are discrete (Siegel et al., 2018) and universal (Barrett, 2006), it may be premature to taut certainty in a field debating the fundamentals.

## Conclusion

Deciphering what another person is feeling is a complex task. Here, we address the role of facial expressions as innate cues and communicative signals, proposing a shift from accuracy measures to judgments in expression authenticity. This includes a comparison of encoding-decoding and affective-induction perspectives to offer insights into the process of emotion expression recognition. We conceive of senders as strategic performers who utilize their full expressive capabilities in social interaction and judges as attempting to infer meaning and intent from emotional displays. To help accelerate progress in the field we encourage researchers to carefully consider theory and methodology in how they operationalize facial expressions.

## Data Availability Statement

The original contributions presented in the study are included in the article/supplementary material, further inquiries can be directed to the corresponding author/s.

## Author Contributions

MZ: conceptualization. MZ and EK: writing and revision. Both authors contributed to the article and approved the submitted version.

## Conflict of Interest

The authors declare that the research was conducted in the absence of any commercial or financial relationships that could be construed as a potential conflict of interest.

## Footnotes

1 High accuracy rates may also result from prototypical and posed expressions typically being used in research (see Krumhuber et al., 2019, 2020; Dupré et al., 2020).

2 If one conceives affect as a knowable and measurable phenomenon, then expression authenticity judgments reflect the ability to detect the emotion being expressed (i.e., it is an objective task, with a correct answer as defined by the researcher). Such a paradigm measures accuracy, i.e., the proximity of the judgment to the target emotion, and precision, i.e., the variability between and within judges and expressions. Alternatively, if one believes that underlying affect is unverifiable, then expression authenticity judgments reflect the perception of different types of emotional displays (i.e., it is a subjective task, considering judgment formation, and variability). Such a paradigm measures only precision.

3 Readers familiar with emotion theories may view encoding-decoding as reflecting the Basic Emotion Theory (BET) and affect-induction as reflecting the Behavioral Ecology View (BECV). The present terminology restricts our reliance on these accounts to certain elements concerned with the conceptualization of facial cues.

## References

[B1] Barrett L. F. (2006). Are emotions natural kinds? *Perspect. Psychol. Sci.* 1 28–58. 26151184 10.1111/j.1745-6916.2006.00003.x

[B2] Barrett L. F. Adolphs R. Marsella S. Martinez A. M. Pollak S. D. (2019). Emotional expressions reconsidered: challenges to inferring emotion from human facial movements. *Psychol. Sci. Public Interest* 20 1–68. 10.1177/1529100619832930 31313636 PMC6640856

[B3] Brysbaert M. Stevens M. (2018). Power analysis and effect size in mixed effects models: a tutorial. *J. Cogn.* 1:9. 10.5334/joc.10 PMC6646942 31517183

[B4] Buck R. Powers S. R. Hull K. S. (2017). Measuring emotional and cognitive empathy using dynamic, naturalistic, and spontaneous emotion displays. *Emotion* 17 1120–1136. 10.1037/emo0000285 28358556

[B5] Calvo M. G. Nummenmaa L. (2015). Perceptual and affective mechanisms in facial expression recognition: an integrative review. *Cogn. Emot.* 30 1081–1106. 10.1080/02699931.2015.1049124 26212348

[B6] Carroll J. M. Russell J. A. (1997). Facial expressions in Hollywood’s portrayal of emotion. *J. Pers. Soc. Psychol.* 72 164–176.

[B7] Coan J. A. Allen J. J. B. (eds). (2007). *Handbook of Emotion Elicitation and Assessment.* New York, NY: Oxford University Press.

[B8] Cohn J. F. Schmidt K. L. (2004). The timing of facial motion in posed and spontaneous smiles. *Int. J. Wavelets Multiresolution Inf. Process.* 02 121–132. 10.1142/s021969130400041x

[B9] Cowie R. Douglas-Cowie E. Cox C. (2005). Beyond emotion archetypes: databases for emotion modelling using neural networks. *Neural Netw.* 18 371–388. 10.1016/j.neunet.2005.03.002 15961273

[B10] Crivelli C. Fridlund A. J. (2018). Facial displays are tools for social influence. *Trends Cogn. Sci.* 22 388–399. 10.1016/j.tics.2018.02.006 29544997

[B11] Crivelli C. Fridlund A. J. (2019). Inside-out: from basic emotions theory to the behavioral ecology view. *J. Nonverbal Behav.* 43 161–194. 10.1007/s10919-019-00294-2

[B12] Crozier W. R. (2010). The puzzle of blushing. *Psychologist* 23 390–393.

[B13] Darwin C. (1872). *The Expression of the Emotions in Man and Animals.* London: John Murray.

[B14] Dawel A. Wright L. Irons J. Dumbleton R. Palermo R. O’Kearney R. (2017). Perceived emotion genuineness: normative ratings for popular facial expression stimuli and the development of perceived-as-genuine and perceived-as-fake sets. *Behav. Res. Methods* 49 1539–1562. 10.3758/s13428-016-0813-2 27928745

[B15] Dezecache G. Mercier H. Scott-Phillips T. C. (2013). An evolutionary approach to emotional communication. *J. Pragmat.* 59 221–233. 10.1016/j.pragma.2013.06.007

[B16] Dupré D. Krumhuber E. G. Küster D. McKeown G. J. (2020). A performance comparison of eight commercially available automatic classifiers for facial affect recognition. *PLoS One* 15:e0231968 10.1371/journal.pone.0231968 PMC7182192 32330178

[B17] Ekman P. (1997). “Expression or communication about emotion,” in *Uniting Psychology and Biology: Integrative Perspectives on Human Development*, eds Segal N. L. Weisfeld G. E. Weisfeld C. C. (Washington, DC: American Psychological Association), 315–338. 10.1037/10242-008

[B18] Ekman P. (2003). *Emotions Revealed: Recognizing Faces and Feelings to Improve Communication and Emotional Life.* New York, NY: Owl Books.

[B19] Ekman P. Friesen W. V. (1971). Constants across cultures in the face and emotion. *J. Pers. Soc. Psychol.* 17 124–129. 10.1037/h0030377 5542557

[B20] Ekman P. Friesen W. V. O’Sullivan M. (1988). Smiles when lying. *J. Pers. Soc. Psychol.* 54:414. 10.1037/0022-3514.54.3.414 3361418

[B21] Ekman P. Levenson R. W. Friesen W. V. (1983). Autonomic nervous system activity distinguishes among emotions. *Science* 221 1208–1210. 10.1126/science.6612338 6612338

[B22] Ekman P. O’Sullivan M. (1991). Who can catch a liar? *Am. Psychol.* 46 913–920. 10.1037/0003-066x.46.9.913 1958011

[B23] Ekman P. Rosenberg E. L. (2005). *What the Face Reveals: Basic and Applied Studies of Spontaneous Expression Using the Facial Action Coding System (FACS).* Oxford: Oxford University Press.

[B24] Fernández-Dols J.-M. Crivelli C. (2013). Emotion and expression: naturalistic studies. *Emot. Rev.* 5 24–29. 10.1177/1754073912457229

[B25] Fiske A. P. (2020). The lexical fallacy in emotion research: mistaking vernacular words for psychological entities. *Psychol. Rev.* 127 95–113. 10.1037/rev0000174 31682141

[B26] Fridlund A. J. (1991). Sociality of solitary smiling: potentiation by an implicit audience. *J. Pers. Soc. Psychol.* 60 229–240. 10.1037/0022-3514.60.2.229

[B27] Gonçalves A. R. Fernandes C. Pasion R. Ferreira-Santos F. Barbosa F. Marques-Teixeira J. (2018). Effects of age on the identification of emotions in facial expressions: a meta-analysis. *PeerJ* 6:e5278. 10.7717/peerj.5278 30065878 PMC6064197

[B28] Gosselin P. Kirouac G. Doré F. Y. (1995). Components and recognition of facial expression in the communication of emotion by actors. *J. Pers. Soc. Psychol.* 68 83–96. 10.1037/0022-3514.68.1.83 7861316

[B29] Gosselin P. Perron M. Beaupré M. (2010). The voluntary control of facial action units in adults. *Emotion* 10 266–271. 10.1037/a0017748 20364903

[B30] Gunnery S. D. Hall J. A. Ruben M. A. (2013). The deliberate duchenne smile: individual differences in expressive control. *J. Nonverbal Behav.* 37 29–41. 10.1007/s10919-012-0139-4

[B31] Gur R. C. Sara R. Hagendoorn M. Marom O. Hughett P. Macy L. (2002). A method for obtaining 3-dimensional facial expressions and its standardization for use in neurocognitive studies. *J. Neurosci. Methods* 115 137–143. 10.1016/s0165-0270(02)00006-7 11992665

[B32] Hall J. A. Andrzejewski S. A. Yopchick J. E. (2009). Psychosocial correlates of interpersonal sensitivity: a meta-analysis. *J. Nonverbal Behav.* 33 149–180. 10.1007/s10919-009-0070-5

[B33] Hess U. Kleck R. E. (1994). The cues decoders use in attempting to differentiate emotion-elicited and posed facial expressions. *Eur. J. Soc. Psychol.* 24 367–381. 10.1002/ejsp.2420240306

[B34] Hurley C. M. Frank M. G. (2011). Executing facial control during deception situations. *J. Nonverbal Behav.* 35 119–131. 10.1007/s10919-010-0102-1

[B35] Izard C. E. (1994). Innate and universal facial expressions: evidence from developmental and cross-cultural research. *Psychol. Bull.* 115 288–299. 10.1037/0033-2909.115.2.288 8165273

[B36] Izard C. E. (2007). Basic emotions, natural kinds, emotion schemas, and a new paradigm. *Perspect. Psychol. Sci.* 2 260–280. 10.1111/j.1745-6916.2007.00044.x 26151969

[B37] Kagan J. (2007). *What is Emotion? History, Measures, and Meanings.* New Haven, CT: Yale University Press.

[B38] Krumhuber E. G. Küster D. Namba S. Shah D. Calvo M. (2019). Emotion recognition from posed and spontaneous dynamic expressions: human observers versus machine analysis. *Emotion* 10.1037/emo0000712 [Epub ahead of print]. 31829721

[B39] Krumhuber E. G. Küster D. Namba S. Skora L. (2020). Human and machine validation of 14 databases of dynamic facial expressions. *Behav. Res. Methods* 10.3758/s13428-020-01443-y [Epub ahead of print]. 32804342 PMC8062366

[B40] Krumhuber E. G. Manstead A. S. R. (2009). Can Duchenne smiles be feigned? New evidence on felt and false smiles. *Emotion* 9 807–820. 10.1037/a0017844 20001124

[B41] Krumhuber E. G. Skora L. Küster D. Fou L. (2017). A review of dynamic datasets for facial expression research. *Emot. Rev.* 9 280–292. 10.1177/1754073916670022

[B42] Levine T. R. Serota K. B. Shulman H. Clare D. D. Park H. S. Shaw A. S. (2011). Sender demeanor: individual differences in sender believability have a powerful impact on deception detection judgments. *Hum. Commun. Res.* 37 377–403. 10.1111/j.1468-2958.2011.01407.x

[B43] Lilienfeld S. O. Sauvigné K. C. Lynn S. J. Cautin R. L. Latzman R. D. Waldman I. D. (2015). Fifty psychological and psychiatric terms to avoid: a list of inaccurate, misleading, misused, ambiguous, and logically confused words and phrases. *Front. Psychol.* 6:1100. 10.3389/fpsyg.2015.01100 26284019 PMC4522609

[B44] McLellan T. Johnston L. Dalrymple-Alford J. Porter R. J. (2010). Sensitivity to genuine versus posed emotion specified in facial displays. *Cogn. Emot.* 24 1277–1292. 10.1080/02699930903306181

[B45] Miller E. J. Krumhuber E. G. Dawel A. (2020). Observers perceive the Duchenne marker as signaling only intensity for sad expressions, not genuine emotion. *Emotion* 10.1037/emo0000772 [Epub ahead of print]. 32718174

[B46] Namba S. Makihara S. Kabir R. S. Miyatani M. Nakao T. (2016). Spontaneous facial expressions are different from posed facial expressions: morphological properties and dynamic sequences. *Curr. Psychol.* 36 593–605. 10.1007/s12144-016-9448-9

[B47] Orlowska A. B. Krumhuber E. G. Rychlowska M. Szarota P. (2018). Dynamics matter: recognition of reward, affiliative, and dominance smiles from dynamic vs. static displays. *Front. Psychol.* 9:938. 10.3389/fpsyg.2018.00938 29942274 PMC6004382

[B48] Ortony A. Turner T. J. (1990). What’s basic about basic emotions? *Psychol. Rev.* 97:315. 10.1037/0033-295x.97.3.315 1669960

[B49] Quigley K. S. Lindquist K. A. Barrett L. F. (2013). “Inducing and measuring emotion and affect: tips, tricks, and secrets,” in *Handbook of Research Methods in Social and Personality Psychology*, eds Reis H. T. Judd C. M. (New York, NY: Cambridge University Press), 220–252. 10.1017/cbo9780511996481.014

[B50] Rinn W. E. (1984). The neuropsychology of facial expression: a review of the neurological and psychological mechanisms for producing facial expressions. *Psychol. Bull.* 95 52–77. 10.1037/0033-2909.95.1.52 6242437

[B51] Sauter D. A. Fischer A. H. (2018). Can perceivers recognise emotions from spontaneous expressions? *Cogn. Emot.* 32 504–515. 10.1080/02699931.2017.1320978 28447544

[B52] Scherer K. R. Bänziger T. (2010). “On the use of actor portrayals in research on emotional expression,” in *A Blueprint for Affective Computing: A Sourcebook and Manual*, eds Scherer K. R. Bänziger T. Roesch E. (Oxford: Oxford University Press), 166–176.

[B53] Shackman A. J. Wager T. D. (2019). The emotional brain: fundamental questions and strategies for future research. *Neurosci. Lett.* 693 68–74. 10.1016/j.neulet.2018.10.012 30473315 PMC6370519

[B54] Siedlecka E. Denson T. F. (2019). Experimental methods for inducing basic emotions: a qualitative review. *Emot. Rev.* 11 87–97. 10.1177/1754073917749016

[B55] Siegel E. H. Sands M. K. Van den Noortgate W. Condon P. Chang Y. Dy J. (2018). Emotion fingerprints or emotion populations? A meta-analytic investigation of autonomic features of emotion categories. *Psychol. Bull.* 144 343–393. 10.1037/bul0000128 29389177 PMC5876074

[B56] Smith D. L. (2004). *Why We Lie: The Evolutionary Roots of Deception and the Unconscious Mind.* New York, NY: St. Martin’s Griffin press.

[B57] Soppe H. J. G. (1988). Age differences in the decoding of affect authenticity and intensity. *J. Nonverbal Behav.* 12 107–119. 10.1007/bf00986929

[B58] Sorensen T. Hohenstein S. Vasishth S. (2016). Bayesian linear mixed models using Stan: a tutorial for psychologists, linguists, and cognitive scientists. *Quant. Methods Psychol.* 12 175–200. 10.20982/tqmp.12.3.p175

[B59] Stanislaw H. Todorov N. (1999). Calculation of signal detection theory measures. *Behav. Res. Methods Instrum. Comput.* 31 137–149. 10.3758/bf03207704 10495845

[B60] Surakka V. Hietanen J. K. (1998). Facial and emotional reactions to Duchenne and non-Duchenne smiles. *Int. J. Psychophysiol.* 29 23–33. 10.1016/s0167-8760(97)00088-3 9641245

[B61] Tomkins S. S. (1962). *Affect Imagery Consciousness: Volume I: The Positive Affects.* New York, NY: Springer publishing company.

[B62] Zloteanu M. (2020). “Reconsidering facial expressions and deception detection,” in *Handbook of Facial Expression of Emotion*, eds Freitas-Magalhaes A. Borod J. (Porto: FEELab Science Books & Leya), 238–284.

[B63] Zloteanu M. Krumhuber E. G. Richardson D. C. (2018). Detecting genuine and deliberate displays of surprise in static and dynamic faces. *Front. Psychol.* 9:1184. 10.3389/fpsyg.2018.01184 30042717 PMC6048358

[B64] Zloteanu M. Krumhuber E. G. Richardson D. C. (2020). Acting surprised: comparing perceptions of different dynamic deliberate expressions. *J. Nonverbal Behav.* 10.1007/s10919-020-00349-9 [Epub ahead of print].

[B65] Zuckerman M. DePaulo B. M. Rosenthal R. (1986). “Humans as deceivers and lie detectors,” in *Nonverbal Communication in the Clinical Context*, eds Blanck P. D. Buck R. Rosenthal R. (University Park, TX: Pennsylvania State University Press), 13–35.

[B66] Zuckerman M. Larrance D. T. Spiegel N. H. Klorman R. (1981). Controlling nonverbal displays: facial expressions and tone of voice. *J. Exp. Soc. Psychol.* 17 506–524. 10.1016/0022-1031(81)90037-8

